# Role of an Ethanolic Extract of *Crotalaria juncea* L. on High-Fat Diet-Induced Hypercholesterolemia

**DOI:** 10.3797/scipharm.1308-08

**Published:** 2014-02-20

**Authors:** Dinakaran Sathis Kumar, Banji David, Avasarala Harani, Bhaskar Vijay

**Affiliations:** 1 Aditya Institute of Pharmaceutical Sciences and Research, Surampalem, Andhra Pradesh, 533437, India.; 2 Jawaharlal Nehru Technical University, Hyderabad, Andhra Pradesh, India.; 3 Nalanda College of Pharmacy, Nalgonda, Andhra Pradesh, 508001, India.; 4 Rangaraya Medical College, Kakinada, Andhra Pradesh, 533003, India.

**Keywords:** *Crotalaria juncea* L., High-fat diet, Lipid profile, Histopathological studies

## Abstract

**Objective:**

To evaluate the antihypercholesterolemic effects of 50 mg/kg BW and 100 mg/kg BW per day of an ethanolic extract of *Crotalaria juncea* Linn (whole plant) by performing *in vivo* studies.

**Methods:**

The effects of oral administration of 50 mg/kg BW and 100 mg/kg BW per day of an ethanolic extract of *Crotalaria juncea* Linn (whole plant) in rats fed with a high-fat diet were investigated by evaluating parameters like food consumption, weight gain, fecal fat excretion, serum and liver lipids, and biochemical profiles as well as by histopathological studies. The results were compared to animals fed with the standard diet and animals fed with a high-fat diet and atorvastatin (10 mg/kg BW).

**Results:**

The animal group administered with the ethanolic extract for 35 days showed decreased levels of TC, LDL, VLDL, TG, HDL+VLDL, VLDL+LDL, LDL/TC, AI, SGOT, SGPT, and elevated levels of HDL, HDL/TC, significantly (p<0.01 & p<0.05) in a dose-dependent manner. The evaluation of liver tissues of the animal groups treated with the herbal extract and standard had shown increased levels of SOD, GSH, and catalase, whereas levels of SGOT, SGPT, total glucose, HMG-CoA, lipase, amylase, and the percentage of malon-dialdehyde were decreased when compared with the high-fat diet-fed rats. Body weight and food intake in the treated groups were significantly lower than that in the model control.

**Conclusion:**

The present study showed that an ethanolic extract of *Crotalaria juncea* L. influences several blood lipid and metabolic parameters in rats, suggesting a potential benefit as an antihypercholesterolemic agent.

## Introduction

Hypercholesteremia, a known risk factor, is considered to be one of the reasons for cardiovascular disease (CAD) and is hence a major cause of premature death globally in many developing and developed countries like India [1] and most European countries, where cardiovascular disease contributes to about 40% of all-cause mortality [2]. It is estimated by the World Health Organization that approximately one-third of all cardiovascular disease worldwide is caused by high cholesterol [3]. Hyperlipidemia is characterized by elevated serum TC, LDL, VLDL, and decreased HDL levels. Hyperlipidemia-associated lipid disorders are found to be responsible for CAD [4], of which hypercholesterolemia and hypertriglyceridemia are closely related to ischemic heart disease [5, 6]. The main aim of treatment in patients with hyperlipidemia is to reduce the risk of developing ischemic heart disease or the occurrence of further cardiovascular or cerebrovascular disease [7]. Hyperlipidemia is classified as primary or secondary based on the complexities associated with the disease, of which anti-lipidemic drugs are used to treat primary disease. The secondary type, originating from diabetes, renal lipid nephrosis, or hypothyroidism, requires the treatment of the actual disease condition rather than simple hyperlipidemia-based treatment [8]. Increased LDL, formed from VLDL due to high fat consumption that adheres to blood vessel walls, can block the normal blood flow resulting in the risk which can be prevented by improving the human diet, which is highly recommended [9]. The treatment of hyperlipidemia involves synthetic hypolipidemic drugs [10] whose consumption may lead to hyperuricemia, diarrhea, nausea, myositis, gastric irritation, flushing, dry skin, and abnormal liver function [11]. Herbal treatment for hypercholesterolemia has been associated with fewer side effects and is relatively cheap, locally available, and some medicinal plants are reported to be effective in reducing the lipid levels [12]. *Crotalaria juncea* L. (Fabaceae), distributed throughout tropical Asia and Africa, is an annually renewable, multi-purpose fiber crop whose extract is used as food as well as medicine by many tribal communities. Generally in the folk and Ayurvedic medicines, it is used as a blood purifier, abortificient, astringent, demulcent, emetic, purgative, and also in the treatment of anemia, impetigo, menorrhagia, and psoriasis [13]. Considering the traditional uses of the plant, the present study was focused on the effects of an extract of the whole plant on serum and liver lipids and other biochemical markers in high-fat diet-fed Sprague Dawley rats.

## Results and Discussion

### Phytochemical Characterization of the Extract

As per our previous chemical screening studies (unpublished data) on CJE, HPLC, HPTLC, and LC-MS/MS, the extract may contain some phenolic compounds like quercetin and its derivatives. Some studies reported [14, 15] that there is evidence for the presence of potentially toxic pyrrolizidine alkaloids (PA) in this plant. As per our previous studies, an LC-MS/MS report of our CJE suggested that the prominent peaks were identified as phenolic compounds based on the MS/MS data. The other unidentified prominent peaks of the MS/MS data were correlated with the known pyrrolizidine alkaloids data. Pyrrolizidine alkaloids could not be detected in the extract.

### Food Intake and Body Weight (Fig. 1, Table 1)

The HF diet groups with or without treatment of CJE did not cause diarrhea during the experiment. The body weight (BW) of the rats in the NC group gradually increased during the 5-week period. Although rats fed with the normal and HF diet continued to show increased body weight and food intake until the end of the study, % body weight reduction was 26.3% (p<0.05) for CJE 50; 30.8% (p<0.01) for CJE 100, and 38.41% (p<0.01) for STD. The percentage reduction in food intake was 16.44% (p<0.05), 16.95% (p<0.05), and 5.91% for CJE50, CJE100, and STD, respectively, and the %food efficiency ratio was 87.18%, 82.3%, and 65.28% (p<0.01) for CJE50, CJE100, and STD, respectively. Food efficiency was increased in the HF group compared with the normal group, but it was reduced in the CJE-treated group. BW and food efficiency were reduced significantly in the atorvastatin-treated group compared to the model control group. In contrast, the BW of the animals fed with the HFD showed a rapid increase, whereas those fed with the HFD and CJE extracts showed a gradual increase in body weight which was significantly less than that of the HFD control in spite of continued and prolonged access to the high-fat diet. The weight loss in the CJE-treated groups resulted in the massive loss of body lipid. Body weight and food efficiency results suggest that CJE may prevent an increase in body weight induced by a high-fat diet; it seemed that low body weight in the herbal-treated groups may be partially due to the loss of appetite.

### Fecal Fat Excretion (Table 2, Fig. 2)

Fecal dry weight in the MC group was significantly higher than in the other groups. In the case of fecal fat excretion, it was significantly higher in the standard-treated group than in the other groups. CJE-treated groups also increased fecal fat excretion in a dose-dependent manner (p<0.05) and the effect was slightly less than that of the standard-treated group, but more than the control groups. From the fecal fat excretion results, it can be considered that atorvastatin- and CJE-treated groups reduced the absorption of cholesterol in the intestine and enhanced the excretion of cholesterol.

### Organ Weight Measurement

There was no significant difference in the relative weight of the lungs, brain, heart, spleen, kidney, liver, and pancreas among the groups. The adipose tissue weight of the MC group was significantly higher than the other groups.

### Serum Analysis (Table 3, Figures 3 and 4)

**Fig. 3:**
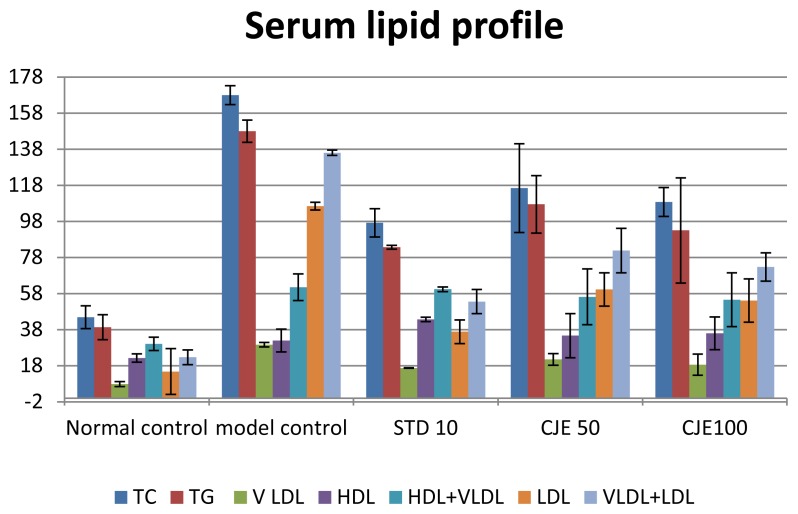
Serum lipid profile

**Fig. 4:**
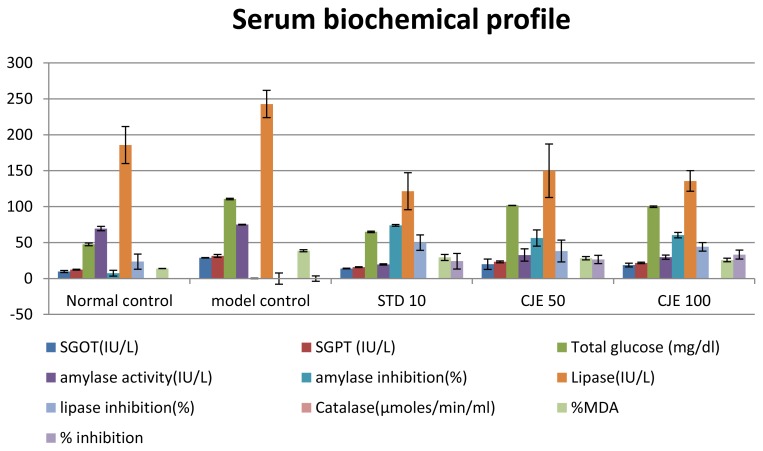
Serum biochemical profile

The lipid and biochemical profiles of the rat serum from all groups are summarized in Table 3. Figures 3 & 4 expressed the effects of CJE on the serum lipid profile and other biochemical parameters. The MC group showed markedly higher serum TC, LDL, VLDL, TG, HDL+VLDL, VLDL+LDL, LDL/TC, and AI levels and lower HDL & HDL/TC levels than the normal control group. Compared with the HFD group, in the CJE-treated groups TC, LDL, VLDL, TG, HDL+VLDL, VLDL+LDL, LDL/TC, and AI were decreased significantly (p<0.01) and HDL and HDL/TC were increased significantly (p<0.01) in a dose-dependent manner. These effects may be due to the low activity of cholesterol biosynthesis enzymes and the low level of lipolysis. The CJE extract supplementation also resulted in a significant attenuation in the level of serum HDL toward the normal control level, which again strengthened the hypolipidemic effect of the extract. Animals treated with atorvastatin showed better results than the CJE-treated groups in all lipid parameters. HDL/TC represents the proportion of the cholesterol component and may provide valid indices for identifying individuals at risk of peripheral arterial diseases. CJE extracts increased the ratio. SGOT, SGPT, plasma glucose, %MDA level, amylase, and lipase levels were increased and catalase activity was decreased in the MC group. SGOT, SGPT, plasma glucose, amylase, lipase, and %MDA (TBARS) were decreased significantly (p<0.05) and catalase activity in plasma was increased in CJE- and atorvastatin-treated groups in a dose-dependent manner after the 35th day of treatment. Atorvastatin-treated groups showed better results than the CJE-treated groups. Plasma glucose reduction in the CJE-treated groups indicated that the CJE extract decreased hyperglycemia in obese rats. In the high-fat diet control, there was a little increment in glucose though it was within the range of 77–150 mg/dl, whereas in the CJE-treated animals, the determined levels were close to the normal range of glucose levels. SGOT and SGPT values were applied to evaluate liver damage in the present study.

### Liver Profile (Table 4, Figures 5 and 6)

In liver tissue, HDL, HDL/TC, SOD, GSH, and catalase were decreased and TC, TG, VLDL, HDL/VLDL, SGOT, SGPT, total glucose, lipase, amylase, HMG-CoA/mevalonate ratio, and the % of malondialdehyde were increased in the model control rats as compared to the normal rats. In the liver, HDL (p<0.05), HDL/TC, SOD, GSH, HMG-CoA/mevalonate ratio, and catalase were increased with the supplementation of CJE extracts and atorvastatin, whereas TC (p<0.05), TG, VLDL, HDL/VLDL, SGOT, SGPT, total glucose (p<0.01), lipase, amylase, and the % of MDA were decreased as compared to the model control in a dose-dependent manner. Lower (p <0.05) liver MDA and higher (p < 0.05) GSH, CAT, and SOD were observed in the NC and CJE extract-treated groups. In the model control groups, the index of the HMG-CoA/mevalonate ratio was decreased, which is responsible for higher cholesterol synthesis. The index of the HMG-CoA/mevalonate ratio was increased in the CJE- and standard-treated groups resulting in decreased cholesterol biosynthesis in the liver. In the groups treated with CJE extracts and the standard, the body weight, parametrial adipose tissue weight, and liver triacylglycerol level were reduced significantly in a dose-dependent manner when compared with the model control group. Theoretically, lipase inhibitors should inhibit fat accumulation in adipose tissue. On the other hand, inhibition of lipase may slow down the clearance of circulating triacylglycerols. CJE extracts increased the fecal lipid content, possibly by inhibiting pancreatic lipase and other gastrointestinal lipases, decreasing the digestibility of dietary fat. The accumulation of triglycerdes in the liver, induced by a high-fat diet, was reduced by the consumption of CJE extracts, possibly because of the inhibition of gastric lipases and the subsequent reduction in intestinal fat absorption and the reduction inlipolysis. Though serum and liver TC and TG were increased in MC, the CJE extract treatment decreased (p<0.05) those values. Conversely, higher (p<0.05) fecal cholesterol outputs were measured in animals treated with herbal extracts as compared to MC. The liver malondialdehyde content was used to represent the liver peroxidation status, while liver GSH, CAT, and SOD were used to evaluate the liver antioxidant capacity in the present study.

### Histopathological Studies

#### Adipose Tissue (Fig. 7)

The adipose tissues used were collected from the subcutaneous region of the rats’ abdomens. The histological appearance of epididymal adipocyte in the MC group was irregular and large in size compared to the other group. However, mild morphological changes and moderate adipocyte size appeared in the CJE-treated groups. These results suggest that the CJE-treated extract supplementation can inhibit lipid accumulation in epididymal adiypocyte tissue. No significant change in the number of nuclei was observed when compared with the model control.

#### Liver (Fig. 8)

The normal control and standard-treated groups’ liver tissues were of normal size. Observation of tissues in the MC rats also showed large vacuoles; fat degeneration; cumulative fatty cysts; moderate vascular congestion; marked dilatation; increased hepatocyte size; distinct enlargement of sinusoids; and sinusoidal dilatation with congestion. The livers of the model control were clearly steatotic. In the CJE-treated group’s livers, dilatated sinusoids, a few bigger-sized heptocytes, and a few lipid droplets were observed, but there was no inflammation, fibrosis, or congestion on them. The standard showed very few lipid droplets and the architecture of the hepatocytes were found to be very similar to that of the normal control group. All treated livers had significantly fewer liver fat depots than the model control, as evaluated by H&E staining. The livers of the treated animals had shown decreased lipid droplets, but there was a slight change in the morphology of the hepatocytes.

#### Aorta (Fig. 9)

The normal control group and standard-treated aortas were normal but in the model control, atherosclerotic plaque was observed on the aorta wall. The histopathology of the aorta of the MC rats indicated lesions with abnormal overlaying endothelia. Observation of the tissues in the MC rats also showed cholesterol deposits and fatty infiltration. In the CJE-treated group, the aortas were similar to the normal control except with fewer inflammatory cells in the vessel wall and congestion.

#### Heart Muscle (Fig. 10)

The normal control and standard-treated groups were normal, but in the model control, fewer lipid droplets were present on the wall. The histopathology of the heart of the MC rats indicated hyaline degeneration in the muscles. Observation of tissues in the CJE-treated rats also showed mild hyaline degeneration in muscles.

The major advantages found in these pathological disorders have been in relation to fat deposition and serum triglycerides. In this way, a statistically significant decrease (P<0.05) has been found in the serum triglyceride levels of animals treated with CJE extracts as compared with MC. The results suggested that the CJE extracts reduced the extent of hypercholesteremia. It may be due to the inhibition of intestinal absorption of cholesterol and the acceleration of catabolism of cholesterol to bile acid. Atorvastatin, which was used as the standard drug in this study, is a HMGR inhibitor which catalyzes the committed step in cholesterol biosynthesis. Statins are HMGR inhibitors that effectively lower serum cholesterol levels and are widely prescribed in the treatment of hypercholesterolemia. Rats treated with atorvastatin showed marked reductions in all serum lipoproteins and increases in HDL levels as compared with the HFD group [16]. Researchers explained that many phenolic compounds have been shown to possess hypolipidemic and antihyper-cholesterolemic activity by increasing the fecal cholesterol excretions and LDL receptor activity [17, 18]. The inferred results were correlated with our previous reports that the phenolic compounds were the major contributors for the antihypercholesterolemic activity of the extract. The present study suggests that both doses of CJE extracts are capable of exerting antihypercholesterolemic effects in high-fat diet-induced rats. Correlations of both studies suggested that phenolic compounds like quercetin and its derivatives may be a reason for the role of *Crotalaria juncea* Linn extract on high-fat diet-induced hypercholesterolemia.

## Conclusion

It can be concluded that the present study supports the folk information regarding the anti-hypercholesteremic activity of *Crotalaria juncea* L. 50 mg/kg BW and 100 mg/kg BW per day of an ethanolic extract of the whole plant showed beneficial anti-hypercholesteremic effects compared with atorvastatin (10 mg/kg BW) or untreated rats. Further studies are required to isolate the active principles from the extract.

## Materials and Methods

### Preparation of the Ethanolic Extract of Crotalaria juncea L. (CJE)

The whole plant was collected from the surrounding areas of the Nalgonda Dist., A.P, India, during November to February (sown in October-November and harvested in February-March) and was identified and authenticated by Mr. A. Lakshma Reddy Retd. Lecturer (Botany), Nagarjuna Government College (Affiliated To Osmania University), Nalgonda. The whole plant material was cleaned, washed, and dried in the shade. After complete drying, the dry plants were kept at room temperature for one week, then powdered (sieved by 80 mesh size), and used for further analysis. The extracts of CJE were prepared by successive soxhlation with various solvents. The shade-dried whole plant powder (250 g) was packed in a thimble kept in the soxhlet apparatus and extraction was allowed to run successively using the solvents, petroleum ether (60 to 80°C), chloroform, and ethanol (95%). Petroleum ether and chloroform were used for defatting. Only the ethanol fraction was concentrated by evaporating the solvent on the water bath and the obtained extracts were weighed. The extraction yield of CJE was found to be 7.88% w/w.

### Preparation of Cholesterol-Rich High-Fat Diet (HFD)

Rat chow diet was supplied by Vyas labs (India). According to data from the supplier it contained crude protein 21.3%, crude fat 4.10%, crude fiber 5.00%, carbohydrate 53%, total ash 7.4%, and minerals and trace elements 5.34% along with other minerals and trace elements like copper 3.20; zinc 35.96; manganese 34.96; and cobalt 0.58 mg/100 g. Vitamins included A 56; D3 0.169; E 0.404; and B complex 5.073 μg/100 g. To the powdered rat chow diet, deoxy cholic acid was added at a ratio of 5 g for 700 g diet and thoroughly mixed. Simultaneously, cholesterol (5 g) was dissolved in 300 g of warm coconut oil. The oil solution was added slowly into the powdered mixture described above to obtain a homogeneous soft cake. This cholesterol-rich (HFD) preparation was molded in the shape of pellets of about 3 g each. The energy level of the prepared high-fat diet was approximately 4996 kcal/kg, whereas the rat chow diet was 3030 kcal/kg.

### Experimental Animals and Group Design

Spraque Dawley rats (5 weeks old) purchased from the Sapthagiri Lab (India) were housed in stainless steel wire-bottomed cages under a 12:12 h light/dark cycle in a temperature- and humidity-controlled room. They were allowed food and water ad libitum. The animal studies were performed in compliance with protocols and policies approved by the Institutional Animal Ethical Committee of Nalanda College of Pharmacy (NCOP), Nalgonda, India (Voucher no: NCOP/IAEC/Approval/22/2010). Rat weights at the beginning of the study ranged from 95 to 120 g after a 1-week time period for adaptation. The rats were randomly divided in to five groups with six animals in each. The doses of 50 mg/kg and 100 mg/kg of CJE, selected as per literature review, and atorvastatin (10 mg/kg) were dissolved in 1% tween 80 solution individually and administered through the oral route by an oral gavage needle. Throughout the experimental period of 35 days, Group I, the normal control group, was fed with the normal rat chow diet, Group II with only the HFD, Group III was treated with the HFD along with standard atorvastatin (10 mg/kg), Group IV and V were administered with the HFD along with CJE 50 mg/kg and 100 mg/kg, respectively. The groups II to V were fed with the high-fat diet to increase the serum lipid levels before the administration of the herbal extract and standard for one week. Dose administration was started from the 7th day. Group I (NC) and Group II (MC) control groups received a 1% vehicle of tween 80 at 10 mL/kg orally. The standard group received atorvastatin at 10 mg/kg orally.

Animals were observed daily for any abnormal physical and behavioral changes or signs of toxicity. Food intake (FT), body weight (BW), mean food efficiency ratios, and fecal fat excretion mass were done for these studies. At the end of the experiment, the rats were fasted for 12 hr and the blood was withdrawn from the retro-orbital plexus of the rats under anesthesia. The blood was centrifuged immediately at 3000 rpm for 15 min to get the plasma samples, and the samples thus obtained were analyzed for the estimation of the lipid profiles like TG, TC, HDL, and LDL, and other biochemical parameters like glucose, SGOT, SGPT, amylase, lipase, catalase, and TBARS by using diagnostic kits (Reckon diagnostic kit, India). All the parameters were estimated using an automatic analyzer (Robonik Touch, version 2.622A). VLDL, LDL, ratios of HDL/TC, AI, and HDL/LDL were calculated to describe serum lipid levels. The animals were sacrificed and the liver was immediately removed and stored at deep freeze conditions until analyzed. Liver tissues were minced and homogenized (10% w/v) in a 0.1 M phosphate buffer (pH 7.4). A part of the homogenized solution was extracted with chloroform–methanol (2:1, v/v, 2 ml). The residue was analyzed with a TG, TC, HDL, LDL kit. The remaining part of the homogenized solution was centrifuged at 5000 × g for 10 min and the resulting supernatant was used for the analysis of SGOT, SGPT, glucose, lipase, and amylase (using the diagnostic kit), catalase, SOD, GSH, and TBARS activity. For HMG-CoA/mevalonate ratio activity, liver tissue was homogenized in a saline arsenate solution. The remaining liver (after the experiment of the hepatic lipid profile), heart, thoracic aorta, and adipose tissues were isolated, cleaned, and then fixed in a buffer solution of 10% neutral-buffered formalin. For the histopathological studies, longitudinal sections of the myocardial tissue, adipose tissue, and thoracic aorta were taken at the macroscopic lesions and the liver sections were cut through the macroscopic lesions including capsules. The sections were further cut to 5 μm thickness and were stained with H&E.

### Statistical Analysis

All the data were subjected to ANOVA (Graph pad Instat Demo software version 3.10). The data shown are mean values and the significance differences were compared by using the Dunnett Multiple comparison test at the P < 0.01 probability level.

## Figures and Tables

**Fig. 1:**
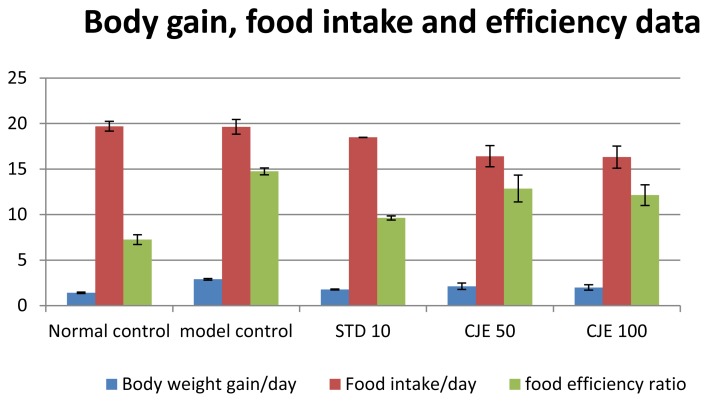
The results of food intake, body weight, and food efficiency ratio

**Fig. 2:**
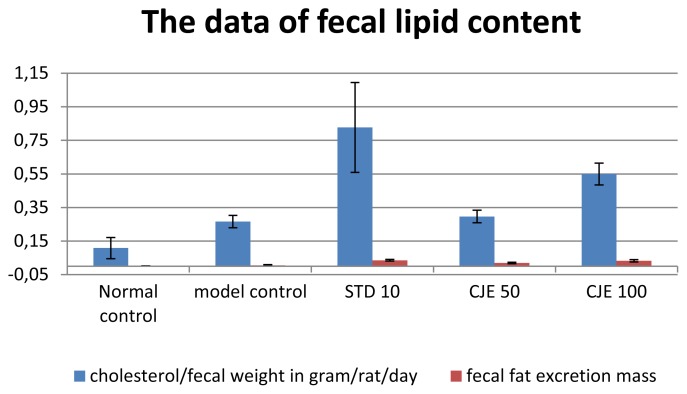
The data of fecal lipid content

**Fig. 5:**
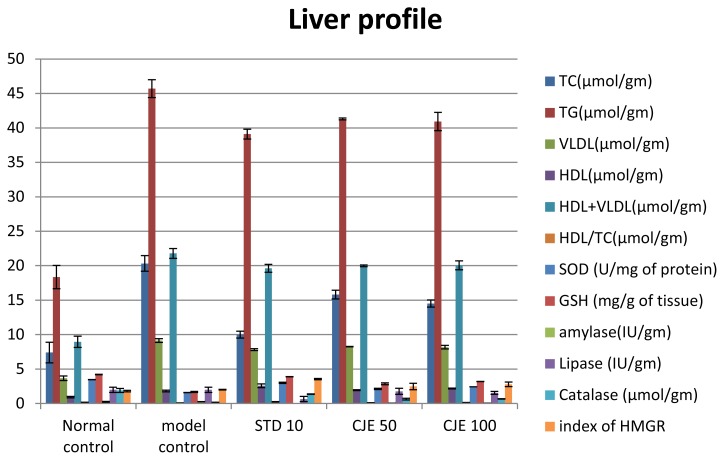
Liver biochemical profile

**Fig. 6:**
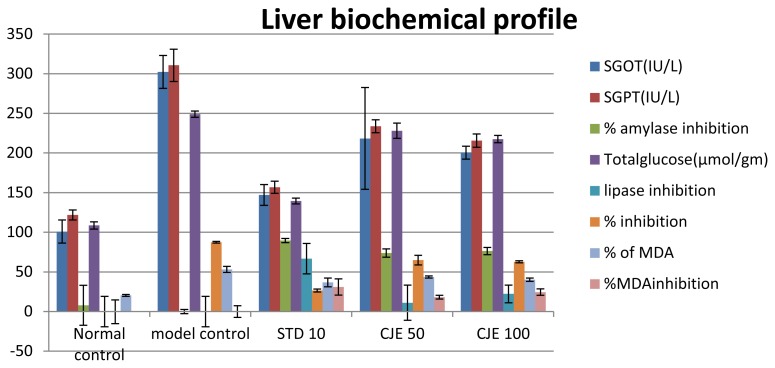
Liver biochemical profile

**Fig. 7:**
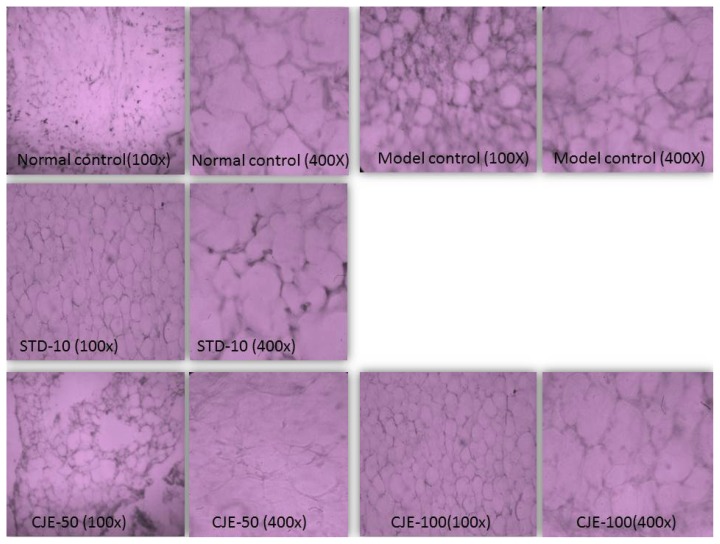
Histopathological studies for adipose tissues

**Fig. 8:**
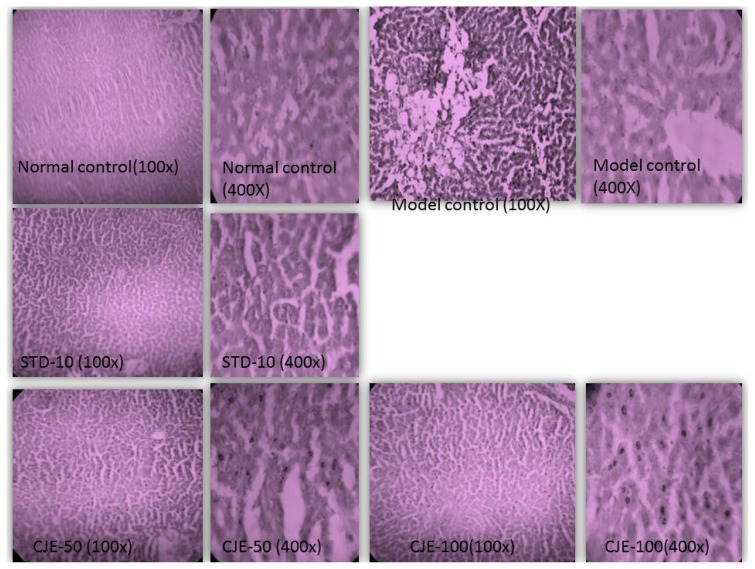
Histopathological studies for liver

**Fig. 9:**
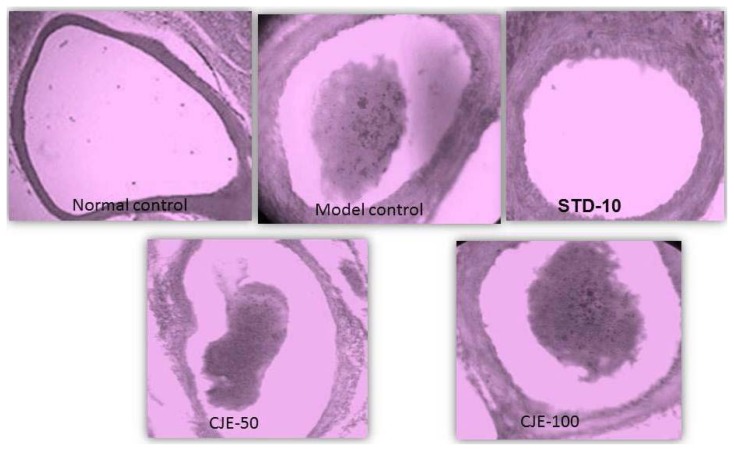
Histopathological studies for aorta

**Fig. 10:**
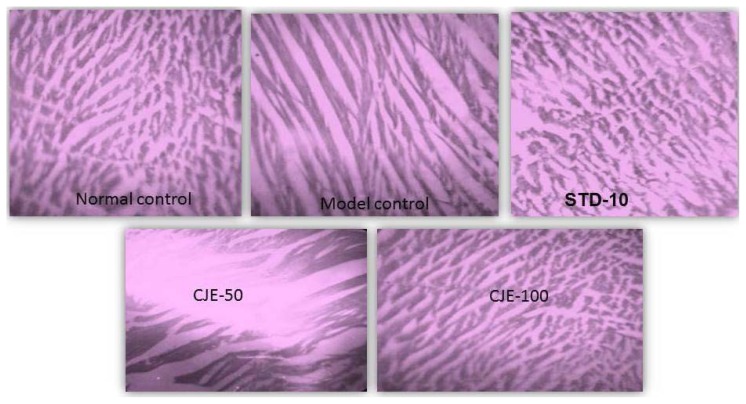
Histopathological studies for heart

**Tab. 1:** The results of food intake, body weight, and food efficiency ratio

	Body weight gain/day^a^	% body weight gain	Food intake/day^a^	% food intake	food efficiency ratio^a^	% food efficiency ratio
Normal control	1.42±0.087^b^		19.71±0.539		7.26±0.533^b^	
model control	2.89±0.084	100	19.65±0.817	100	14.75±0.383	100
STD 10	1.78±0.042^b^	61.59	18.49±0.019	94.09	9.63±0.221^b^	65.28
CJE 50	2.13±0.346^c^	73.7	16.42±1.152^c^	83.56	12.86±1.473	87.18
CJE 100	2±0.296^b^	69.2	16.32±1.214^c^	83.05	12.14±1.13	82.3

a mean of 6 ±standard error mean;

b P<0.01;

c P<0.05 value was found to be significant as compared to the control group.

**Tab. 2:** The data of fecal lipid content

	cholesterol/fecal weight in gram/rat/day^a^	fecal fat excretion mass^a^	fecal weight^a^
Normal control	0.1087±0.0632	0.0015±0.0015	3.742±0.0247
model control	0.2665±0.0366	0.0062±0.0031	3.953±0.0207
STD 10	0.8272±0.2673	0.0361±0.0050^c^	3.847±0.0688
CJE 50	0.2965±0.0376	0.0203±0.0041	3.840±0.031
CJE 100	0.5493±0.0654	0.0328±0.0071^c^	3.793±0.024

a mean of 6 ±standard error mean;

b P<0.01;

c P<0.05 value was found to be significant as compared to the control group.

**Tab. 3:** The data of serum analysis

(mg/dl)	TC^a^	TG^a^	VLDL^a^	HDL^a^	HDL+ VLDL^a^	LDL^a^	VLDL+ LDL^a^	AIa	HDL/ TC^a^	LDL/ TC^a^
**Normal control**	44.90 ±6.25^b^	39.39 ±6.9^b^	7.87 ±1.38^b^	22.24 ±2.31	30.12 ±3.66	14.78 ±2.71^b^	22.66 ±4.07^b^	1.00 ±0.10^c^	0.50 ±0.02^b^	0.32 ±0.02^b^
**Model control**	167.94 ±5.24	148.01 ±6.14	29.60 ±1.22	31.93 ±6.33	61.54 ±7.36	106.39 ±2.16	136.00 ±1.46	4.72 ±1.17	0.18 ±0.03	0.63 ±0.03
**STD 10**	97.22 ±7.87^b^	83.68 ±1.11^c^	16.73 ±0.22^c^	43.63 ±1.21	60.37 ±1.34	36.84 ±6.53^b^	53.58 ±6.67^b^	1.22 ±0.12*	0.45 ±0.02^b^	0.37 ±0.03^b^
**CJE 50**	116.44 ±24.55^c^	107.46 ±15.90	21.49 ±3.18	34.67 ±12.29	56.16 ±15.45	60.28 ±9.19^b^	81.77 ±12.28^b^	3.08 ±1.03	0.274 ±0.057	0.534 ±0.040
**CJE 100**	108.68 ±8.01^b^	93.01 ±29.18^c^	18.60± 5.84^c^	35.94 ±9.09	54.54 ±14.93	54.14 ±12.02^b^	72.74 ±7.79^b^	2.46 ±0.95	0.328 ±0.071	0.504 ±0.117

**(mg/dl)**	**SGOT (IU/L)**^a^	**SGPT (IU/L)**^a^	**Total glucose (mg/dl)**^a^	**Amylasea**		**Lipase**		**Catalase (μmoles/min/ml)**^a^	**% MDA**^a^	**% inhib.**^a^

				**activity (IU/L)**	**inhib. (%)**	**(IU/L)**^a^	**inhib. (%)**^a^			

**Normal control**	9.75 ±1.35^b^	12.38 ±0.65^b^	47.60 ±1.91^b^	69.46 ±2.99^b^	7.46 ±3.98^b^	185.71 ±25.75	23.53 ±10.61	0.014 ±0.007^c^	13.83 ±0.14^b^	
**Model control**	28.88 ±0.38	31.51 ±1.95	110.68 ±1.06	75.07 ±0.36^b^	−0.012 ±0.482^b^	242.86 ±18.90	−0.003 ±7.782	0.004 ±0	38.60 ±1.44	−0.010 ±3.736
**STD 10**	13.88 ±0.38^c^	15.76 ±0.65^b^	64.95 ±1.08^b^	19.54 ±0.83	73.97 ±1.10	121.43 ±25.75	50.00 ±10.61	0.034 ±0.001^b^	29.30 ±4.18^c^	24.11 ±10.82^c^
**CJE 50**	19.88 ±7.13	23.26 ±1.35	101.65 ±0.26	32.74 ±8.51	56.38 ±11.33	150 ±37.12	38.23 ±15.28	0.012 ±0	28.34 ±2.26^c^	26.59 ±5.86^c^
**CJE 100**	18.76 ±2.63	21.76 ±0.99^c^	99.93 ±0.96	29.67 ±2.81	60.48 ±3.74	135.71 ±14.29	44.12 ±5.88	0.015 ±0^c^	25.74 ±2.42^b^	33.33 ±6.27^b^

a mean of 6 ±standard error mean;

b P<0.01;

c P<0.05 value was found to be significant as compared to the control group.

**Tab. 4:** The data of liver profiles

	TC	TG	VLDL	HDL	HDL +VLDL	HDL /TC	SGOT (IU/L)^a^	SGPT (IU/L)^a^	SOD (U/mg of protein)^a^	GSH (mg/g of tissue)^a^

	(μmol/gm)^a^									
**Normal control**	7.40 ±1.5^b^	18.36 ±1.69^b^	3.67 ±0.34^b^	0.94 ±0.109^b^	8.96 ±0.82^b^	0.14 ±0.04	100.90 ±14.53^b^	121.91 ±6.31^b^	3.48 ±0.02	4.21 ±0.02
**Model control**	20.32 ±1.13	45.70 ±1.3	9.14 ±0.27	1.81 ±0.12	21.79 ±0.71	0.09 ±0.01	302.34 ±20.72	310.59 ±20.47	1.59 ±0.01	1.7 ±0.078
**STD 10**	10.01 ±0.50^b^	39.10 ±0.72^b^	7.82 ±0.14^b^	2.57 ±0.27^b^	19.63 ±0.58	0.25 ±0.03^b^	147.04 ±13.16^b^	156.79 ±7.5^b^	3 ±0.08	3.89 ±0.02
**CJE 50**	15.83 ±0.63^c^	41.29 ±0.14	8.26 ±0.03	1.94 ±0.06	19.98 ±0.12	0.123 ±0.002	218.32 ±64.23	233.69 ±8.18	2.12 ±0.08	2.87 ±0.13
**CJE 100**	14.52 ±0.52^b^	40.92 ±1.34	8.18 ±0.27	2.19 ±0.06	20.06 ±0.64	0.151 ±0.002	200.31 ±8.12	215.69 ±8.46^c^	2.43 ±0.01	3.2 ±0.02

	**Total glucose (μmol/ gm)**^a^	**Amy-lase (IU/ gm)**^a^	**% inhib.**^a^	**Lipase (IU/ gm)**^a^	**Lipase inhib.**^a^	**Cata-lase (μmol/ gm)**^a^	**% inhib.**^a^	**index of HMGR**^a^	**% MDA**^a^	**% inhib.**^a^

**Normal control**	108.68 ±4.58^b^	0.25 ±0.06^b^	7.84 ±25.11^b^	2 ±0.38	0 ±19.24	1.91 ±0.29^b^	−0.23 ±15.03 b	1.80 ±0.11^b^	20.39 ±1.121^b^	
**Model control**	248.90 ±3.95	0.27 ±0.007	−0.05 ±2.63	2 ±0.38	0 ±19.24	0.18 ±0.02	87.48 ±1.02	2.03 ±0.04	53.11 ±3.85	−0.008 ±7.25
**STD 10**	139.53 ±3.66^b^	0.02 ±0.00^b^	89.46 ±2.63^b^	0.66 ±0.38	66.66 ±19.24	1.38 ±0.03^b^	26.55 ±1.77^b^	3.54 ±0.09^b^	36.68 ±5.46^b^	30.92 ±10.28^b^
**CJE 50**	228.09 ±9.59	0.072 ±0.014	73.67 ±5.27	1.78 ±0.44	11.11 ±22.22	0.632 ±0.120	64.91 ±6.09	2.48 ±0.46	43.53 ±1.26	18.04 ±2.36
**CJE 100**	217.52 ±4.58^b^	0.065 ±0.013	76.30 ±4.56	1.56 ±0.22	22.22± 11.11	0.673 ±0.025	62.81 ±1.27	2.79 ±0.33^c^	40.11 ±2.151^c^	24.48 ±4.05^c^

a mean of 6 ±standard error mean;

b P<0.01;

c P<0.05 value was found to be significant as compared to the control group.

## Abbreviations

AI: atherogenic index
ANOVA: analysis of variance
BW: body weight
CAD: atherosclerotic coronary artery disease
CJE: ethanolic extract of *Crotalaria juncea* Linn
GSH: reduced glutathione
H & E: haematoxylin and eosin
HDL: high density lipoprotein
HFD: high-fat diet (HF, high-fat)
HMG-CoA: 3-hydroxy-3-methylglutaryl–coenzyme A
HMGR: HMG-CoA reductase
LCAT: lecithin cholesterol acyl transferase
LDL: low density lipoprotein
MC: model high-fat diet control
MDA: malondialdehyde
NC: normal diet control
PL: pancreatic lipase
SGOT: serum glutamate oxaloacetate transaminase
SGPT: serum glutamate pyruvate transaminase
SOD: superoxide dismutase
STD: atorvastatin
TBARS: thiobarbituric acid reactive substances
TC: total cholesterol
TG: triglyceride
VLDL: very low density lipoprotein
WHO: World Health Organisation
